# Identification and validation of immune cells and hub genes alterations in recurrent implantation failure: A GEO data mining study

**DOI:** 10.3389/fgene.2022.1094978

**Published:** 2023-01-09

**Authors:** Liangcheng Yu, Lu Wang, Lijin Wang, Song Yan, Shuqiang Chen, Qian Xu, Danjie Su, Xiaohong Wang

**Affiliations:** ^1^ Department of Gynecology and Obstetrics, Tangdu Hospital, Air Force Medical University, Xi’an, China; ^2^ Department of Cardiology, Tangdu Hospital, Air Force Medical University, Xi’an, China

**Keywords:** recurrent implantation failure, γδT cells, activated memory CD4 T cells, diagnostic biomarker genes, competing endogenous RNA regulatory network

## Abstract

**Introduction:** Recurrent implantation failure (RIF) is a distressing problem in assisted reproductive technology (ART). Immunity plays a vital role in recurrent implantation failure (RIF) occurrence and development, but its underlying mechanism still needs to be fully elucidated. Through bioinformatics analysis, this study aims to identify the RIF-associated immune cell types and immune-related genes.

**Methods:** The differentially expressed genes (DEGs) were screened based on RIF-associated Gene Expression Omnibus (GEO) datasets. Then, the enrichment analysis and protein-protein interaction (PPI) analysis were conducted with the DEGs. The RIF-associated immune cell types were clarified by combining single sample gene set enrichment analysis (ssGSEA) and CIBERSORT. Differentially expressed immune cell types-related modules were identified by weighted gene co-expression network analysis (WGCNA) and local maximal quasi-clique merger (lmQCM) analysis. The overlapping genes between DEGs and genes contained by modules mentioned above were delineated as candidate hub genes and validated in another two external datasets. Finally, the microRNAs (miRNAs) and long non-coding RNAs (lncRNAs) that interacted with hub genes were predicted, and the competing endogenous RNA (ceRNA) regulatory network was structured.

**Results:** In the present study, we collected 324 DEGs between RIF and the control group, which functions were mainly enriched in immune-related signaling pathways. Regarding differential cell types, the RIF group had a higher proportion of activated memory CD4 T cells and a lower proportion of γδ T cells in the endometrial tissue. Finally, three immune-related hub genes (ALOX5AP, SLC7A7, and PTGS2) were identified and verified to effectively discriminate RIF from control individuals with a specificity rate of 90.8% and a sensitivity rate of 90.8%. In addition, we constructed a key ceRNA network that is expected to mediate molecular mechanisms in RIF.

**Conclusion:** Our study identified the intricate correlation between immune cell types and RIF and provided new immune-related hub genes that offer promising diagnostic and therapeutic targets for RIF.

## 1 Introduction

As a recognized global public health issue, infertility is estimated to affect at least 186 million people (Inhorn and Patrizio, 2015). Encouragingly, assisted reproductive technologies (ARTs) are considered safe medical interventions, with approximately eight million children born (Faddy et al., 2018; Fauser, 2019). However, recurrent implantation failure (RIF), which generally refers to a woman’s inability to conceive after at least three transfers of quality embryos *in vitro* fertilization (IVF) (Coughlan et al., 2013), has emerged as a challenging clinical dilemma in ART, frustrating clinicians and patients alike (Hill, 2021). Approximately 10%-15% of couples experienced RIF during *in vitro* fertilization-embryo transfer (IVF-ET) (Busnelli et al., 2020). The underlying mechanisms of RIF are complex and related to various factors, such as the maternal immune system, embryonic and parental genetics, anatomical characteristics, hematological factors, reproductive tract microbiome, and endocrine milieu (Franasiak et al., 2021). Numerous studies have suggested that immune factors, especially the immune microenvironment of the endometrium, play a crucial role in the process of pregnancy (Larsen et al., 2013; Sebastian-Leon et al., 2018; Robertson et al., 2022). Both flow cytometry and tissue immunostaining studies showed that human decidual leukocytes in the first trimester are predominantly natural killer (NK) cells (∼70%) and macrophages (∼20%) (Trundley and Moffett, 2004; Bulmer et al., 2010). The proportion of T cells is highly variable (10%–20%), while dendritic cells (DCs), B cells, and NKT cells are rare (Erlebacher, 2013). The tolerance of decidual T cells to fetal alloantigens (especially HLA- C allotypes) expressed in the extravillous trophectoderm has been reported to be critical for a successful pregnancy (Moffett and Shreeve, 2022). Nevertheless, the function of decidual T cells is currently largely unknown (Erlebacher, 2013). In humans, decidual changes occur to some extent throughout the entire endometrium during the secretory phase of the menstrual cycle, even in the absence of implantation (Erlebacher, 2013). Thus, the endometrium taken from the mid-luteal phase in this study can characterize the immune cellular changes in the decidua of early pregnancy.

In recent years, with the development and widespread use of high throughput “omics” approaches, bioinformatics analysis can be applied to mine these published data to identify novel genes and biomarkers for many diseases (Segundo-Val and Sanz-Lozano, 2016; Xu et al., 2022). For instance, Lin et al. used bioinformatics analysis to identify AXL, SLC7A11, and ubiquilin 1 (UBQLN1) as essential oxidative stress-related genes with predictive value for the development of RIF (Lin and Lin, 2022). Although there are many studies using bioinformatics approaches to study differentially expressed genes (DEGs) and immune infiltration in RIF and recurrent pregnancy loss (RPL) (Ticconi et al., 2019; Mrozikiewicz et al., 2021), few studies on RIF have applied deep bioinformatics analysis, such as WGCNA and CIBERSORT, which limits insights for a more comprehensive elucidation of RIF etiology.

Our study aims to explore immune cell types and hub genes that may be involved in RIF occurrence through multiple transcriptional microarray datasets by applying deep bioinformatics analysis. This study will contribute to understanding the mechanisms of immune dysfunction in RIF and provide therapeutic insights.

## 2 Materials and methods

### 2.1 Microarray data acquisition

Gene expression profiles of RIF were screened from the GEO (http://www.ncbi.nlm.nih.gov/geo) database. Inclusion criteria were as follows: 1) *Homo sapiens* expression profiling by the array; 2) samples were endometrium of RIF patients or control individuals (CON) during the window of implantation; 3) datasets contained ten or more samples with at least five patients in each group, and 4) RIF patients and fertile controls were included in one experiment. This study ultimately included four datasets sed on the above selection criteria, including GSE111974, GSE92324, GSE26787, and GSE71835. Details of all data are shown in Table 1.

**TABLE 1:** Basic information of selected datasets.

GEO	Platform	Samples size	Average age (years)	Attribute	Country/References
		RIF/CON	RIF/CON		
GSE111974	GPL17077	24/24	33/31	Test	Turkey (Bastu et al., 2019)
GSE92324	GPL10558	10/8	33/26	Test	India (Pathare et al., 2017)
GSE26787	GPL570	5/5	33/32	Validation	France (Ledee et al., 2011)
GSE71835	GPL10558	6/6	31/25	Validation	India (Pathare et al., 2017)

### 2.2 Data preprocessing and study design

We merged GSE111974 and GSE92324 microarray data as test datasets. Specifically, the first step is to convert the series matrix file from gene probe IDs to gene symbol codes, averaged for the case of one gene corresponding to multiple probes. The second step is to remove the batch effect, we first used limma’s removeBatchEffect function (Ritchie et al., 2015), yet it failed to eliminate the batch effect between GSE111974 and GSE92324 (Supplementary Figure S1). We then used sva’s combat function (Leek et al., 2012) to eliminate the batch effect between the datasets, and Supplementary Figure S2 showed that the batch effect was successfully eliminated for the merged data. This may be explained by the fact that the removeBatchEffect function removes known batch effects from the data (Ritchie et al., 2015), while sva package not only removes known batch effects but also adjusts for other potentially unwanted sources of variation in the data for subsequent analysis (Leek et al., 2012).

The final step is to normalize the expression values through the limma package to have a similar distribution in a set of arrays. Here, we have drawn up a flow chart of the analysis process **(** Supplementary Figure S3 **)**.

### 2.3 DEGs selection and enrichment analysis

The differentially expressed genes (DEGs) between RIF patients and CON were selected by using the limma package with the |Log2FC (fold change) | > 1 and adjusted *p*-value < 0.05. Analyses of Gene Ontology (GO) and Kyoto Encyclopedia of Genes and Genomes (KEGG) for DEGs were performed by the clusterprofiler package (Yu et al., 2012), the significantly different GO terms were determined by thresholds adjusted *p*-value < 0.05, and KEGG pathways with a *p*-value <0.05 was selected. In addition, to discover candidate genes sets or pathways that likely contribute to RIF, gene set enrichment analysis (GSEA) (Subramanian et al., 2005) was performed by the clusterprofiler package to scrutinize the gene expression profile at an entire level, and C5 (ontology gene sets) was chosen for functional enrichment analyses. The normalized enrichment score (|NES| >1), *p*-value <0.05, and adjusted *p*-value <0.05 were set as threshold criteria.

### 2.4 Evaluation of immune cell types alteration

In this study, 28 immune cell types and associated 782 marker gene signatures were first obtained from two previously reported studies (Supplementary Table S1) (Barbie et al., 2009; Charoentong et al., 2017). Then, the abundance of these immune cell types in endometrial samples was calculated by the single sample gene set enrichment analysis (ssGSEA) method based on the genomic variance analysis (GSVA) algorithm. Recently, the application of ssGSEA in deconvolution of bulk gene expression data has been widely performed (Zhao et al., 2021; Liu et al., 2022).

CIBERSORT is another method to calculate cell composition based on expression profiles. In the present study, we used CIBERSORT to assess immune cell infiltration in endometrial tissue between RIF and CON. The leukocyte signature matrix (LM22) was used as a reference expression signature with 1,000 permutations (Zhou et al., 2021). LM22 signature matrix contains 22 infiltrating immune cell components and the corresponding 547 signature genes (Supplementary Table S2) (Newman et al., 2015). Then the Wilcoxon test was conducted to determine significant differences in immune cell types between RIF and CON.

### 2.5 Gene co-expression network construction and modules selection

The weighted gene co-expression network analysis (WGCNA) is an algorithm that can find modules of a co-expressed gene with high biological significance (Langfelder and Horvath, 2008). In this study, to reduce the whole network’s computation size but maintain a scale-free topological network, we selected the genes in the top 75% based on the magnitude of the variance. Then we entered them into the WGCNA package in R to identify the gene modules associated with significantly altered immune cell types. Briefly, genes with similar expression patterns were assigned to co-expression modules by weighted correlated adjacency matrices and clustering analysis. Firstly, the weighted adjacency matrix is constructed by calculating an appropriate soft threshold *β* that satisfies the criteria for a scale-free network. Afterward, the weighted adjacency matrix was converted to a topological overlap matrix (TOM), and the corresponding dissimilarity degree (1-TOM) was generated. Then, module identification was performed using the dynamic tree-cutting method, and modules with differences less than 0.25 were merged. In addition, the relationship between module eigengene values and immune cell types was assessed by Pearson correlation.

Additionally, we performed the local maximal quasi-clique merger (lmQCM) to network mining (Zhang and Huang, 2014) based on the merged matrix by the lmQCM package in R. The parameters for lmQCM were set as follows: gamma = 0.55, t = 1, lambda = 1, beta = 0.4, and minimum cluster size = 10. The lmQCM is a weighted network mining algorithm that detects weak quasilinear modules in a weighted graph and applies it to the discovery of functional gene clusters. The algorithm is characterized by a greedy approach using hierarchical clustering that does not allow overlap between modules but allows genes to be shared between multiple modules. This is in accordance with the fact that genes are often involved in multiple biological processes (Bichindaritz et al., 2021).

Among the weighted network modules constructed from WGCNA and lmQCM, we selected the module with the highest or lowest correlation coefficient as the specific module associated with the differentially expressed immune cell types. This research defined the modules most relevant to γδ T cells and activated memory CD4 T cells as crucial modules. The genes contained in the crucial modules were defined as differentially expressed immune cell types-related genes (DE ICTRGs).

### 2.6 Identification and enrichment analysis of hub genes

The overlapping genes between DEGs and DE ICTRGs were identified with the Venn online platform (http://bioinformatics.psb.ugent.be/webtools/Venn/) and defined as potential hub genes. If too many genes were overlapping, they would be further filtered according to the protein-protein interaction information from STRING (https://string-db.org) with confidence scores ≥0.4. The interaction file (string_interactions.tsv) was downloaded. Subsequently, ten algorithms of cytoHubba (Chin et al., 2014) in Cytoscape 3.9.0 (Shannon et al., 2003) were conducted to score each node gene, namely, MCC (Maximal Clique Centrality), MNC (Maximum Neighborhood Component), Degree, EPC (Edge Percolated Component), BottleNeck, EcCentricity, Closeness, Radiality, Betweenness, and Stress. Lastly, the ten node genes with the highest scores for each algorithm were examined for hub genes using the UpSet package in R. Furthermore, GeneMANIA (http://genemania.org) analyses were performed to examine protein and gene interactions, pathways, co-expression, co-localization, and protein domain similarities (Franz et al., 2018).

### 2.7 Validation and efficacy evaluation of hub genes

To further validate the accuracy and reliability of the hub genes selected from test datasets, two external datasets, GSE26787 and GSE71835 microarray data, were downloaded from the GEO database and combined using the approach mentioned above. Firstly, the expression of hub genes was extracted from the test sets and validation sets and analyzed by the Wilcoxon test, with a *p*-value of <0.05 defined as statistical significance. Then, we constructed a prediction model using the differentially expressed hub genes by the generalized multivariate regression with the test sets. Finally, we calculated the sensitivity rate and specificity rate of the model, Receiver Operating Characteristic (ROC) analysis was also performed to detect the Area Under the Curve (AUC).

### 2.8 Construction of competing endogenous RNA (ceRNA)-regulating network

The multiMiR package in R is a comprehensive collection of predicted and validated miRNA–target interactions and their associations with diseases and drugs (Ru et al., 2014), including 14 databases. In the present study, we selected three databases (DIANA-microT, miRanda, and TargetScan) to predict the targeted miRNAs of the hub genes. The top 35% of miRNAs in the prediction scores of these three databases were intersected. The intersection was used for subsequent analysis. For all long non-coding RNAs (lncRNAs)-miRNA interaction data were acquired in the starbase database (https://starbase.sysu.edu.cn/) (Li et al., 2014), and the target lncRNAs were filtered according to clipExpNum >4. Eventually, the established network was visualized by Cytoscape software.

### 2.9 Statistical analysis

In this study, all data analysis and visualization were performed using R software (version 4.0.5; https://www.r-project.org/) with appropriate packages. *p*-value <0.05 was considered significant.

## 3 Results

### 3.1 Data pre-processing

We downloaded GSE111974 and GSE26787 from the GEO database as test datasets, including 34 RIF and 32 normal endometrial tissues. Box plots and principal component analysis shows the data before batch correction (A, C, and E) and after batch correction (B, D, and F) (Supplementary Figure S2), which indicates that the batch effect was successfully eliminated from the combined data.

### 3.2 DEGs identification and enrichment analysis

**FIGURE 1:**
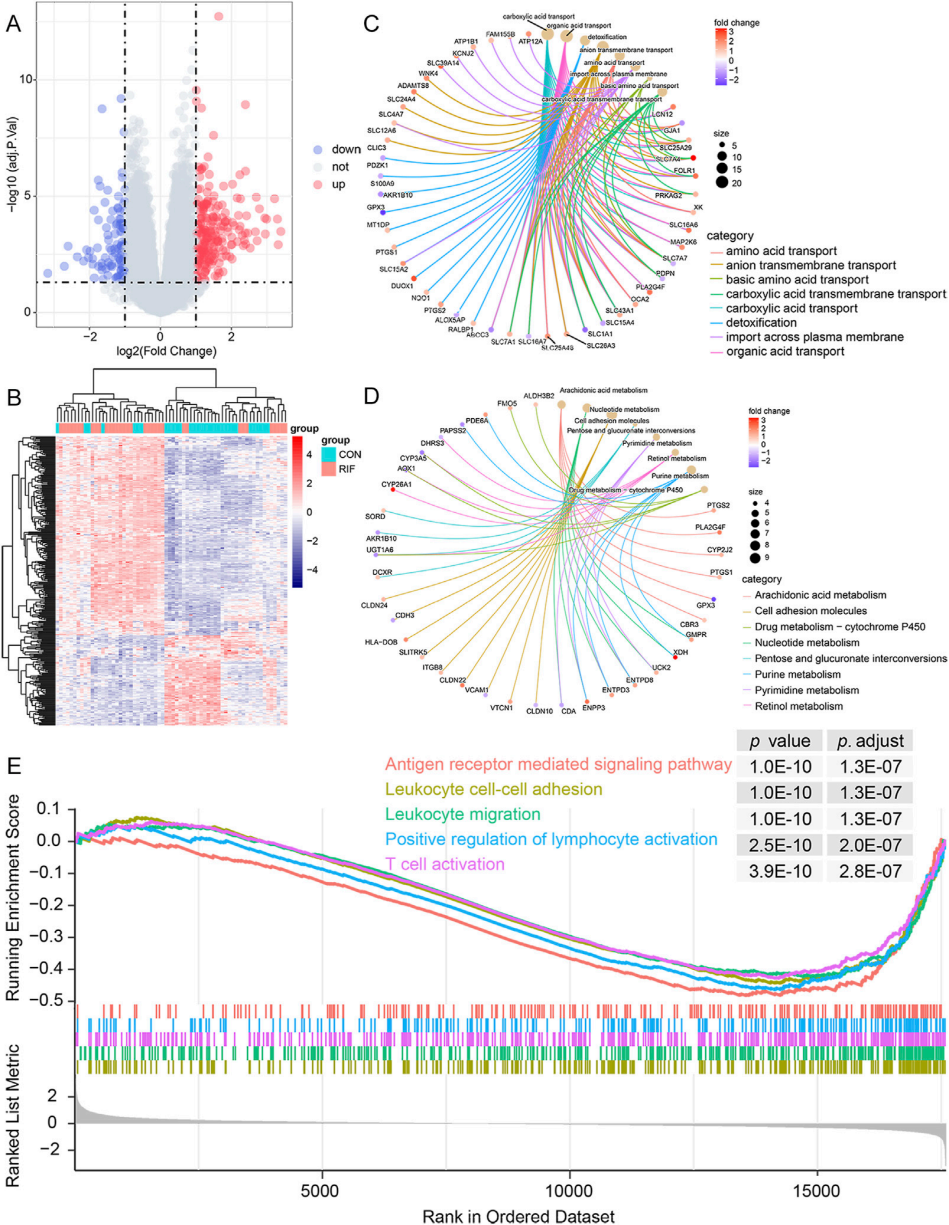
Functional enrichment analysis of DEGs. **(A)**, volcano plot of DEGs between RIF and CON individuals. There were 223 up-regulated and 101 down-regulated genes in the RIF group. Besides, two vertical dashed lines represent Log2 (fold change) at -1 and 1; the horizontal dashed line represents the adjusted *p*-value at 0.05. **(B)**, the heatmap of DEGs between the RIF and CON groups. **(C)**, the top eight GO terms in the biological process were shown in the functional enrichment analysis of DEGs. Adjusted *p*-value <0.05 was identified as significantly changed GOs. **(D)**, top eight KEGG pathway analysis was conducted on DEGs, and *p*-value <0.05 was selected as a significantly changed KEGG pathway. **(E)**, GSEA plot showing the top 5 enriched immune-related gene sets in the RIF and CON groups based on the adjusted *p*-value. Abbreviations: DEGs, differentially expressed genes; RIF, recurrent implantation failure; CON, control individuals; GO, Gene Ontology; KEGG, Kyoto Encyclopedia of Genes and Genomes; GSEA, gene set enrichment analysis.

A total of 324 DEGs were identified (223 significantly up-regulated genes and 101 significantly down-regulated genes) (Figures 1A,B; Supplementary Table S3). Then, the DEGs were subjected to GO and KEGG pathway enrichment analyses. The GO enrichment revealed that these DEGs were mainly associated with carboxylic acid transport, organic acid transport, and detoxification in the biological process. Regarding cellular components, the genes were primarily enriched in the apical plasma membrane, apical part of the cell, and collagen-containing extracellular matrix. As for molecular function, the genes were enriched primarily in anion transmembrane transporter activity, organic anion transmembrane transporter activity, and active transmembrane transporter activity (Figure 1C; Supplementary Table S4). Likewise, the KEGG analysis demonstrated that these DEGs are relevant to immune pathways such as TNF signaling pathway, Leukocyte transendothelial migration, and NF-kappa B signaling pathway (Figure 1D; Supplementary Table S4). In addition, the GSEA results showed that 385 gene sets were significant at an adjusted *p*-value <0.05, and most of the enriched gene sets were related to various immune responses (Supplementary Table S5). Figure 1E shows the five most enriched immune-related gene sets based on the adjusted *p*-value. These were related to leukocyte cell-cell adhesion, leukocyte migration, T cell activation, positive regulation of lymphocyte activation, and antigen receptor-mediated signaling pathway.

### 3.3 Alterations of immune cells in the endometrium of RIF and CON

**FIGURE 2:**
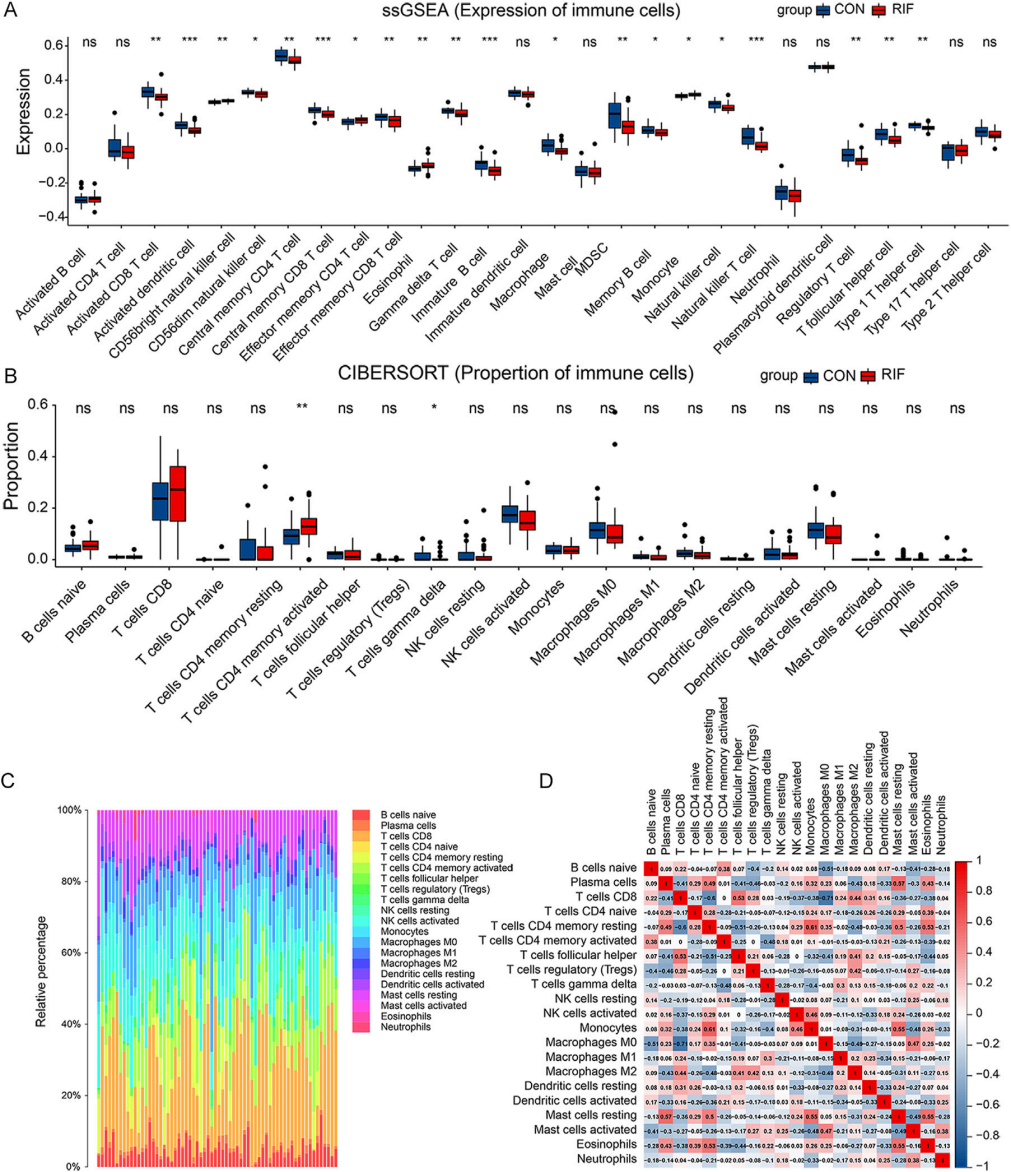
Immune cell alteration of RIF patients. **(A)**, the result of the cell-specific marker of immune cell types expressed in the two groups. **(B)**, the proportional distribution of diverse immune cell types between the two groups. **(C)**, histogram presenting immune cell type changes. **(D)**, the correlation matrix of the changes in the number of 22 immune cell populations in the endometrial tissue of RIF. Red: positive correlation; blue: negative correlation. (ns, no significance, *p < 0.05, **p < 0.01, ***p < 0.001).

Next, we explored immune cell changes in the test set. First, ssGSEA identified 16 immune cell subtypes, including activated CD8 T cells, activated dendritic cells, CD56 dim natural killer cells, central memory CD4 T cells, central memory CD8 T cells, and γδ T cells. Their cell-specific marker genes were lower expressed in the RIF group (Figure 2A). Conversely, the specific marker genes of three immune cells (CD56 bright natural killer cells, effector memory CD4 T cells, and eosinophils) were expressed at higher levels in the RIF group. Furthermore, compared with the CON group, CIBERSORT analysis demonstrated that activated memory CD4 T cells had statistically higher abundance. In comparison, γδT cells had statistically lower abundance in the RIF group (Figure 2B), which is consistent with the results of ssGSEA. The above results indicated that γδT cells and activated memory CD4 T cells were the significantly altered cell types in the RIF group. In addition, the constituency of the 22 immune cell types in each sample was plotted as a histogram by performing CIBERSORT (Figure 2C). Meanwhile, the correlation between these 22 immune cell types in endometrial tissue from the RIF group was calculated (Figure 2D). Figure 2D shows a significant positive correlation between memory resting CD4 T cells and monocytes (R = 0.61). Also, plasma cells were positively correlated with resting mast cells (R = 0.57). In contrast, CD8 T cells were negatively correlated with macrophage M0 (R = -0.71). Likewise, γδT cells were negatively associated with activated memory CD4 T cells (R = -0.48). Nevertheless, the association mentioned above of immune cells was attenuated to null in CON (Supplementary Figure S4).

### 3.4 Gene co-expression network construction and modules selection

**FIGURE 3:**
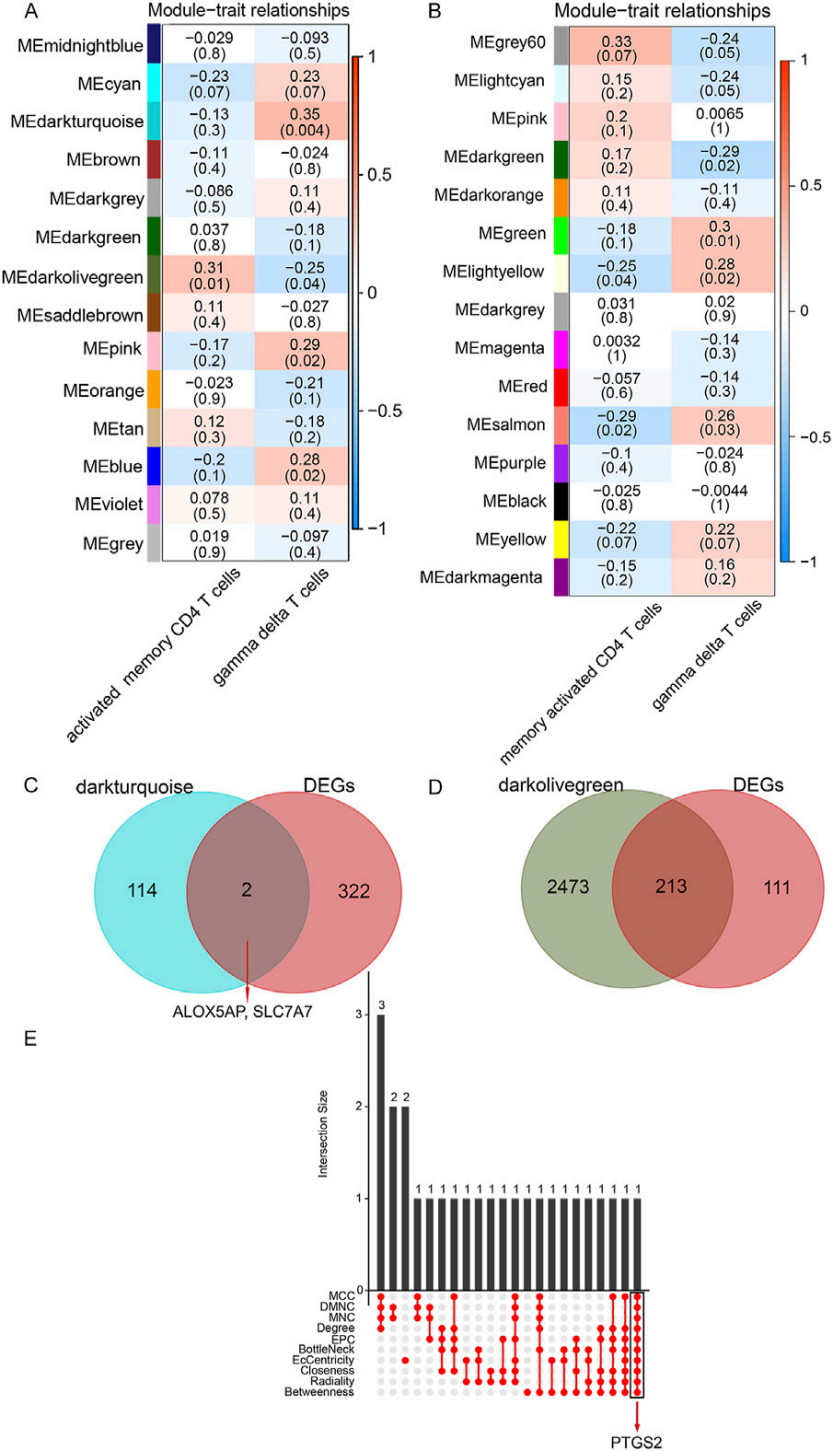
Identification of crucial modules and common DEGs. **(A)**, Module–trait relationships in WGCNA modules. **(B)**, Module–trait relationships in lmQCM modules. The number in the first row in each cell represents the Pearson correlation coefficient, and the *p*-value of the corresponding module trait is exhibited in parentheses. The color of each cell indicates the degree of correlation. (Red indicates a positive correlation, and blue indicates a negative correlation). **(C)**, Venn diagram of shared genes between DEGs and γδ T cells-associated genes. **(D)**, Venn diagram of shared genes between DEGs and activated memory CD4 T cells -associated genes. **(E)**, Ten algorithms were utilized to screen key genes from the shared genes between DEGs and activated memory CD4 T cells -associated genes.

In WGCNA analysis, the 13,194 genes in the top 75% based on the magnitude of the variance were included in the WGCNA analysis, and the soft power of *β* = 10 (scale-free *R* ^2^ > 0.85) was determined as soft-thresholding to acquire co-expressed gene modules (Supplementary Figures S5A, B). Then, dynamic hybrid cuts were conducted to construct hierarchical clustering trees by dividing the dendrogram at relevant transition points (Supplementary Figure S5C). Of which, single genes were represented as tree leaves, multiple genes with analogous expression data were presented as branches of the dendrogram tree, and branches containing similarly expressed genes were considered gene modules. Similarly, another differentially expressed immune cell types co-expression network was also constructed by lmQCM analysis. Ultimately, we got 14 WGCNA modules (Figure 3A) and 15 lmQCM modules (Figure 3B).

Among these, the dark olive-green module (correlation = 0.31, *p*-value = 0.01) was the most relevant module identified by WGCNA for activated memory CD4 cells, and the salmon module (correlation = -0.29, *p*-value = 0.02) was the most relevant module identified by lmQCM for activated memory CD4 cells. Thus the dark olive-green module was selected as the activated memory CD4 cell-associated key module for further analysis. Similarly, the dark turquoise module (correlation = 0.35, *p*-value = 0.004) was the most relevant module identified by WGCNA for γδT cells, and the green module (correlation = 0.3, *p*-value = 0.01) was the most relevant module identified by lmQCM for γδT cells. Thus the dark turquoise module was selected as the key module associated with the γδT cells module for further analysis. In this study, we defined genes in these two modules most relevant to γδ T cells and activated memory CD4 T cells as DE ICTRGs.

### 3.5 Identification and enrichment of hub genes

**FIGURE 4:**
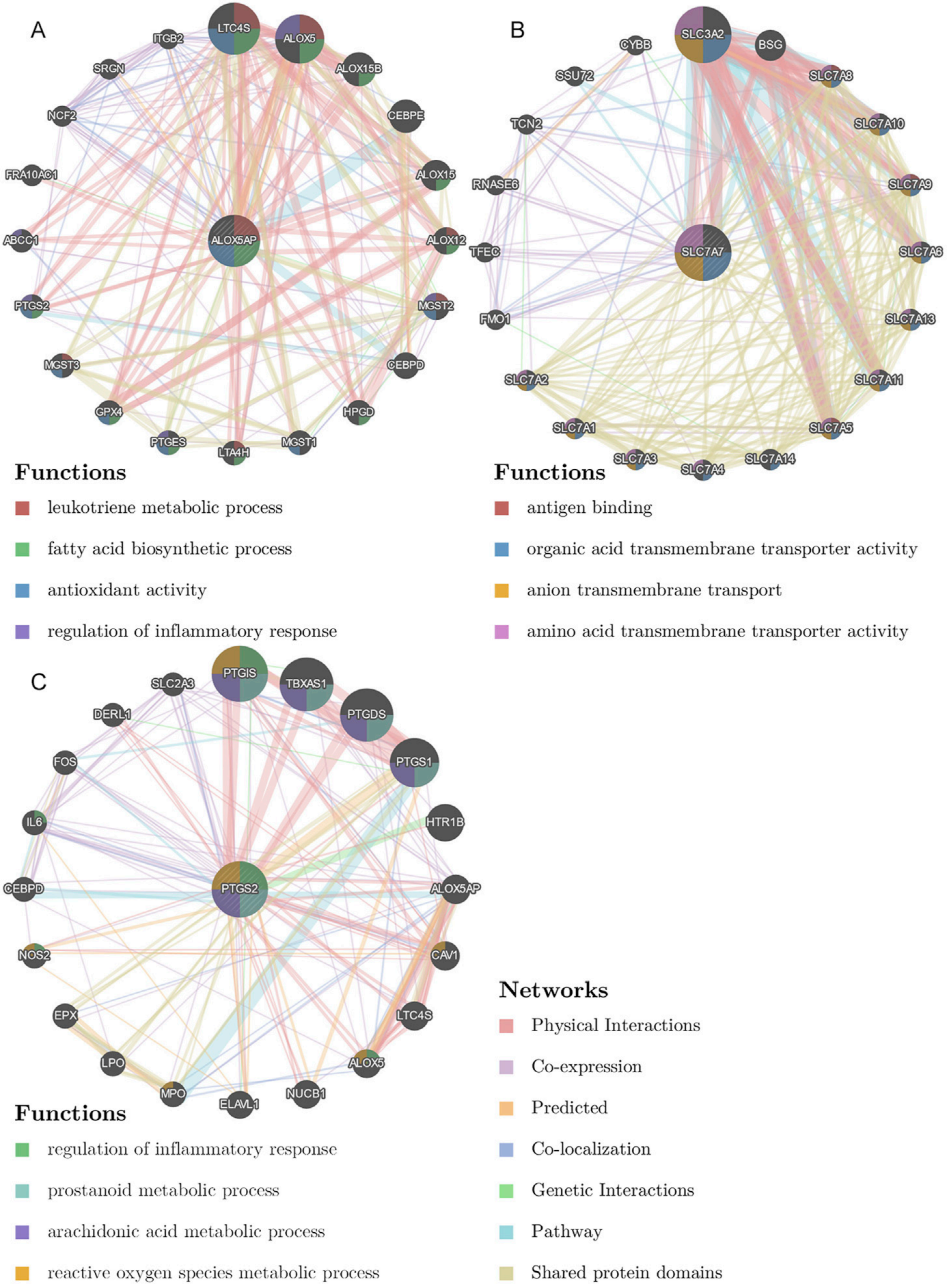
The gene-gene interaction network for hub genes were analyzed using the GeneMANIA database. **(A)**, The gene-gene interaction network of ALOX5AP analyzed by GeneMANIA. **(B)**, The gene-gene interaction network of SLC7A7 analyzed by GeneMANIA. **(C)**, The gene-gene interaction network of PTGS2 analyzed by GeneMANIA. The 20 most frequently changed neighboring genes are shown. The predicted genes are located in the outer circle, and the hub genes are in the inner circle.

We collected shared genes from DE ICTRGs and DEGs using a Venn diagram. It turned out that two overlapping genes between γδ T cells-associated genes and DEGs (ALOX5AP, SLC7A7) (Figure 3C), and 213 overlapping genes between activated memory CD4 T cells-associated genes and DEGs (Figure 3D) (Supplementary Table S6). Considering there were too many overlapping genes in Figure 3D, the PPI network was constructed for 213 genes and filtered by cytoHubba in Cytoscape. The results of the CytoHubba were listed in Supplementary Table S7, and PTGS2 was determined as the hub gene (Figure 3E). Finally, we explored three hub genes (ALOX5AP, SLC7A7, and PTGS2) and their 20 interacting genes using the GeneMANIA database (Figure 4). The network illustrated that these genes were relevant to immune processes such as the leukotriene metabolic process, antigen binding, and regulation of inflammatory response.

### 3.6 Validation and efficacy evaluation of hub genes

**FIGURE 5:**
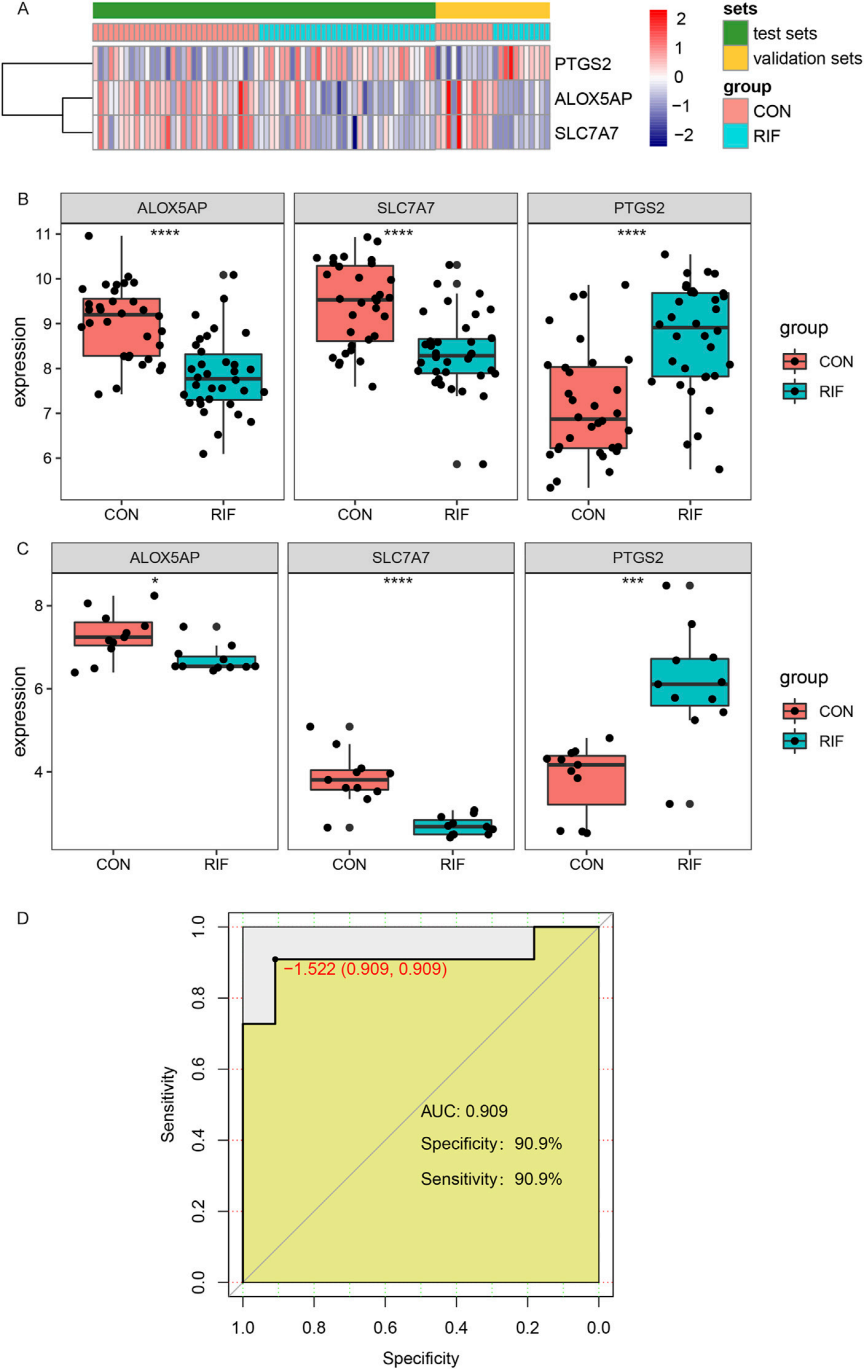
Validation of hub genes and ROC curves of the hub genes between the RIF and CON group. **(A)**, The Expression of three hub genes was presented by heatmap in test and validation sets. **(B)**, the expressions of ALOX5AP, SLC7A7, and PTGS2 in test sets (GSE111974 and GSE92324). **(C)**, the expressions of ALOX5AP, SLC7A7, and PTGS2 in validation sets (GSE26787 and GSE71835). **(D)**, The ROC curve of the combined three hub genes in predicting RIF. Abbreviations: ROC, receiver operating characteristic curves. *p < 0.05, **p < 0.01, ***p < 0.001, ****p < 0.0001.

For validating the identified hub genes, another two datasets, GSE26787 and GSE71835, were merged after removing the batch effect (Supplementary Figure S6), including 11 RIF and 11 normal endometrial tissues. The expression levels of ALOX5AP, SLC7A7, and PTGS2 were presented in the heatmap (Figure 5A). As shown in Figures 5B,C, in both the test set and validation set, the expression levels of ALOX5AP and SLC7A7 were significantly decreased, and PTGS2 was increased dramatically in the RIF group (*p* < 0.05). In addition, we used the datasets GSE26787 and GSE71835 as validation sets to investigate the predictive effect of hub genes for RIF. Encouragingly, in the validation set, the prediction model showed a specificity of 90.8% and sensitivity of 90.8%, and the ROC analysis showed that the AUC was 0.908 (Figure 5D).

### 3.7 Construction of the ceRNA-regulating network

**FIGURE 6:**
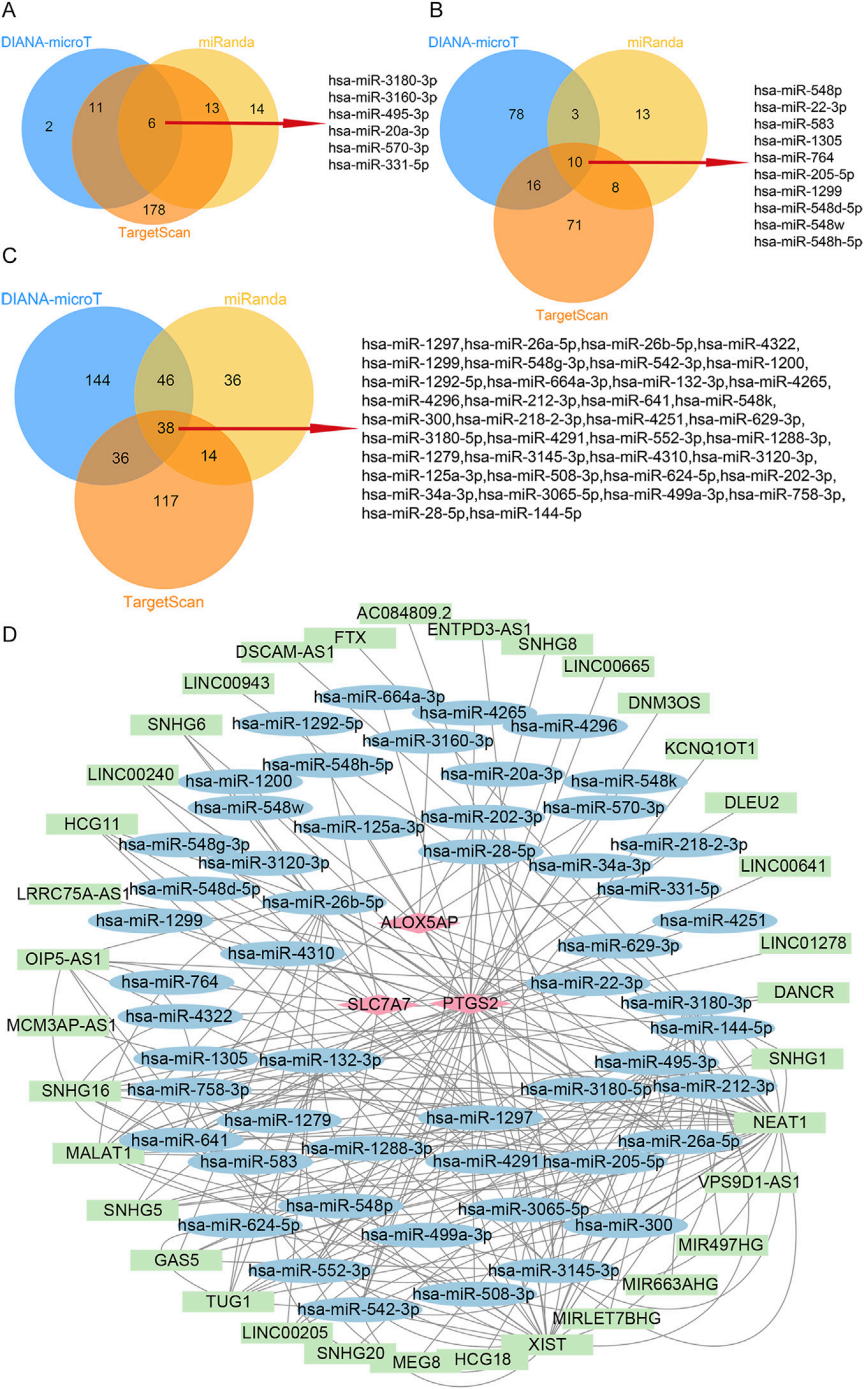
ceRNA-regulating networks. **(A)**, The Venn diagram indicates six miRNAs that interacted with ALOX5AP from the DIANA-microT, miRanda, and TargetScan. **(B)**, The Venn diagram indicates ten miRNAs interacting with SLC7A7 from the DIANA-microT, miRanda, and TargetScan. **(C)**, The Venn diagram indicates 38 miRNAs interacting with PTGS2 from the DIANA-microT, miRanda, and TargetScan. **(D)**, the red diamond represents the protein-coding genes, the blue circle represents miRNAs, and the green rectangle represents lncRNAs. The black lines indicate the interaction of lncRNA–miRNA–mRNA. Abbreviations: ceRNA, competing endogenous RNAs;miRNAs, microRNAs; lncRNAs, long non-coding RNAs.

To explore possible interactions between lncRNAs, miRNAs, and mRNA in RIF, we structured a ceRNA regulatory network. In the present study, we collected 53 miRNAs, including hsa-miR-3180-3p, hsa-miR-548p, and hsa-miR-1297 (Figures 6A–C). Next, we mapped the abovementioned 53 miRNAs into the starbase database and searched for the target lncRNAs. As a result, 35 lncRNAs that interacted with 19 of the 53 miRNAs in the starbase database were selected (Supplementary Table S8). Eventually, the ceRNA regulatory network was structured, and the visualization was carried out in the CytoScape software (Figure 6D).

## 4 Discussion

As a complex clinical disease in the IVF-ET cycle, RIF brings a tremendous burden to patients and treatment challenges to physicians. Studies have shown that the endometrial factor is one of the main factors contributing to RIF (Timeva et al., 2014). Therefore, identifying essential dysregulated genes in the endometrium of RIF is clinically relevant for the prevention and diagnosis of RIF. Most of these GO-enriched terms of DEGs are related to carboxylic acid transport, amino acid transport, etc. Zeng et al. found that dietary Arginine supplementation in early pregnancy in rats enhances embryo implantation by stimulating PI3K/PKB/mTOR/NO signaling pathway (Zeng et al., 2013). There are two amino acid transport systems associated with mouse oocytes or with preimplantation of mouse embryos: 1) sodium-independent L-transport system; and 2) sodium-dependent A-transport system (Colonna and Mangia, 1983; Colonna et al., 1984). These studies suggest an essential role for amino acids in pre- and post-implantation of the placenta and embryo development. In addition, based on the results of KEGG and GSEA enrichment, we can conclude that the immune response is related to the pathogenesis of implantation failure.

The definitive etiology of RIF is poorly understood in almost 50% of cases and yet could be closely linked to abnormalities in maternal immune regulation (Azizi et al., 2019), especially related to the immune-tolerant microenvironment at the maternal-fetal interface (Ander et al., 2019). The primary immune cells that establish and maintain immune tolerance in the maternal-fetal interface are maternal decidual natural killer (NK) cells, macrophages, and T cells (Mor et al., 2011). To make the results more robust, we took the intersection of the results of CIBERSORT and ssGSEA in the present study. We found that the mid-luteal phase endometrium of the RIF group had a lower proportion of γδ T cells and a higher proportion of activated memory CD4 T cells compared with the control group. Regrettably, we did not observe significant changes in the proportion of NK cells in the current study. The results of the present study corroborate that in a systematic review that included 22 articles suggesting there was no significant difference in the percentage of peripheral or endometrial NK cells in infertile women compared with fertile controls (Seshadri and Sunkara, 2014). However, some reports demonstrated that women with RIF or RPL have a higher percentage of endometrial NK cells and blood NK cells than controls (Sacks et al., 2012; Santillan et al., 2015; Zhu et al., 2017). The non-consensus definition of NK cells can explain the apparent discrepancy in the results of these studies (Kolanska et al., 2019). Moreover, in the presence of sex hormones, the concentration of endometrial immune cells fluctuates during the menstrual cycle, and their proliferation and activation depend on locally secreted factors (Wira et al., 2015). Third, peripheral blood and endometrium-producing immune cells are heterogeneous (Daussy et al., 2014), and the phenotypes of peripheral blood and endometrial NK cells differ (Moffett-King, 2002; Mekinian et al., 2016).

Both implantation and placenta formation has been reported to be pro-inflammatory processes involving multiple cytokines (Ramhorst et al., 2006; Orsi, 2008). During the peri-implantation period, γδ T cells could express TNF-α and IFN-γ (Fan et al., 2011), two common pro-inflammatory cytokines, which may exhibit anti-infection activity against foreign antigens in pregnancy. Similarly, the same results have been found in mice studies (Arck et al., 1997), suggesting that γδ T cells play an essential role in early pregnancy, especially during embryo implantation. In the present study, we found that the abundance of γδ T cells in the RIF group significantly decreased. To better understand the potential role of γδ T cells in the pathophysiological process of RIF, we further identified its closely related hub genes ALOX5AP and SLC7A7. Notably, the expression levels of ALOX5AP and SLC7A7 were significantly reduced in the RIF group. ALOX5AP is a crucial enzyme required for the production of the inflammatory mediator leukotrienes (LTs) *via* the 5-lipoxygenase (5-LOX) pathway (Mashima and Okuyama, 2015), and the leukotriene metabolite LBT4 is required for γδ T cell migration during inflammatory reactions (Costa et al., 2010). Therefore, downregulation of ALOX5AP Expression may lead to a decrease in leukotriene production, adversely affecting γδ T cell migration and ultimately leading to embryo implantation failure. As shown in Figure 4C, SLC7A5-13 and SLC7A15 form the L-type amino acid transporter protein (LAT) family, Cibrian et al. found that CD69, a typical marker of γδ T cells, expressed by γδ T cells regulates cellular activity by controlling the uptake of tryptophan by LAT1 (Cibrian et al., 2016; Cibrian and Sanchez-Madrid, 2017). Thus, when the expression of LAT family genes is abnormal, the activity of γδ T cells is also affected, which is consistent with the results observed in this study. In addition, in this study, we observed that the abundance of activated memory CD4 T cells was significantly higher in the endometrium of the RIF group, and the expression of its associated hub gene PTGS2 was also significantly upregulated in the RIF group. PTGS2 encodes cyclooxygenase-2 (COX-2), the rate-limiting enzyme for PGE2 compounds (Murakami and Kudo, 2004). Napolitani et al. demonstrate that PGE2 can act directly on memory CD4 T cells leading to an increase in IL-17 production (Napolitani et al., 2009). Therefore, when PTGS2 is overexpressed, IL-17 levels are elevated, and the increased IL-17 expression is reported to participate in maternal immune rejection of the fetus (Wang et al., 2019), leading to implantation failure.

In recent years, growing studies suggested that lncRNAs- and miRNAs-mediated molecular mechanisms were associated with the occurrence of RIF. The present study predicted a total of 53 miRNAs associated with hub genes. Among them, Tochigi et al. demonstrated that miR-542-3p overexpression inhibits the induction of major decidual marker genes, including IGFBP1, WNT4, and PRL, which suggested that miR-542-3p plays an important role in endometrial decidualization by regulating the expression of major decidual marker genes (Tochigi et al., 2017). In the present study, we found that miR-542-3p interacted with the hub gene PTGS2, suggesting that miR-542-3p may affect endometrial decidualization by regulating PTGS2 expression. Moreover, endometrial decidualization represents a crucial step for the successful implantation of the embryo, indicating that dysregulation of miR-542-3p may cause implantation failure. In addition, we also predicted 35 lncRNAs associated with hub genes, many of which have been shown to play a critical role in the pregnancy process. For example, Shi et al. confirmed that LncRNA MALAT1 promotes decidualization of human endometrial stromal cells (hESCs) to maintain a successful pregnancy (Shi et al., 2022), and downregulated MALAT1 relates to RPL (Wang et al., 2018). This study showed that MALAT1 interacted with the key gene SLC7A7 through miR-205-5p, miR-22-3p, and similarly, MALAT1 also interacts with the key gene PTGS2 through miR-1297, miR-26a-5p, miR-26b-5p, miR-28-5p, miR-3145-3p, miR-508–3. These lncRNAs and mRNAs could compete for the same miRNA response elements (MREs) to mutually regulate (Sen et al., 2014). Herein, we constructed the ceRNA-regulating network to clarify the interaction between lncRNA and miRNA and its potential role in regulating RIF-related gene expression. Although our data may not validate all predicted lncRNAs and miRNAs, it could provide insights for subsequent studies.

Some limitations should be acknowledged in the current study. First, the present study was a retrospective analysis of publicly available datasets. As additional clinical information about the patients cannot be obtained, we cannot exclude that other factors may have confounded our analysis. Second, we have not validated this study’s results through laboratory experiments, and subsequent confirmatory experiments *in vivo* and *in vitro* are required.

In summary, our study not only offered insights into the landscape of immune cells and identified some hub genes for RIF but also constructed the ceRNA-regulating network that contributed to the understanding of the pathophysiological process of RIF by bioinformatics analysis, which provided the potential diagnostic and therapeutic targets of RIF.

## Data Availability

Publicly available datasets were analyzed in this study. This data can be found here: http://www.ncbi.nlm.nih.gov/geo.
