# Supplementary figures and images for: Identification and validation of immune cells and hub genes alterations in recurrent implantation failure: A GEO data mining study
Source: Front Genet. 2023 Jan 9;13:1094978. doi: 10.3389/fgene.2022.1094978 (PMC9868458; doi:10.3389/fgene.2022.1094978)

A

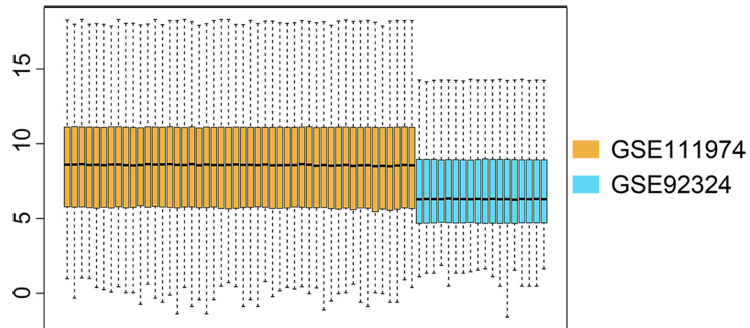

B

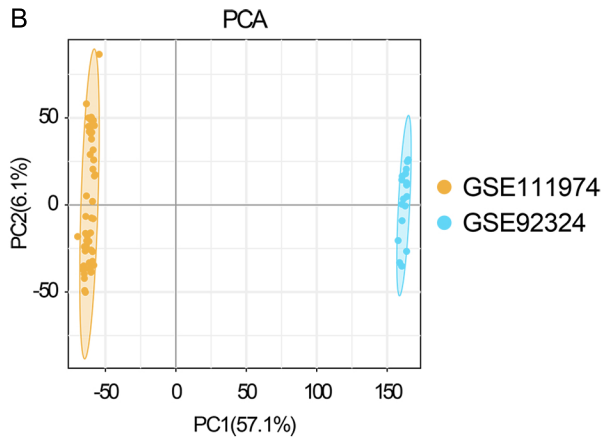

C

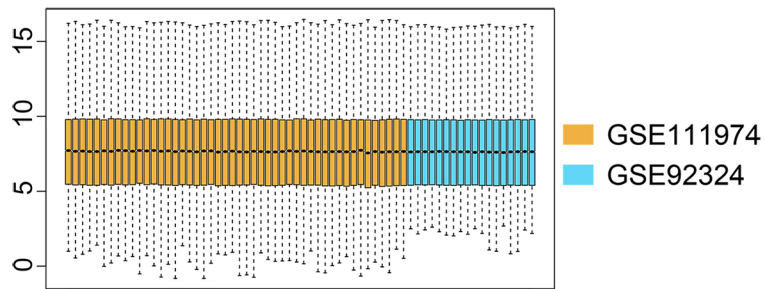

D

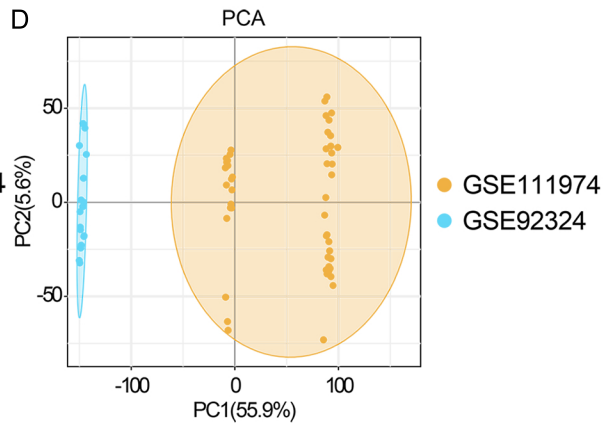

Supplement: Supplementary file 1 [file DataSheet1.ZIP › Figure S1.pdf]

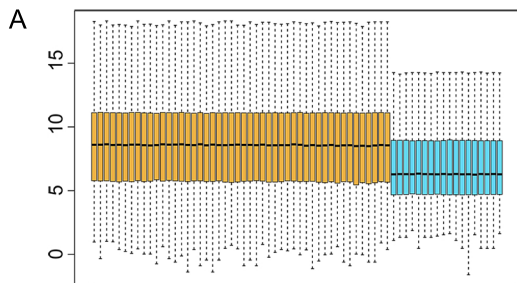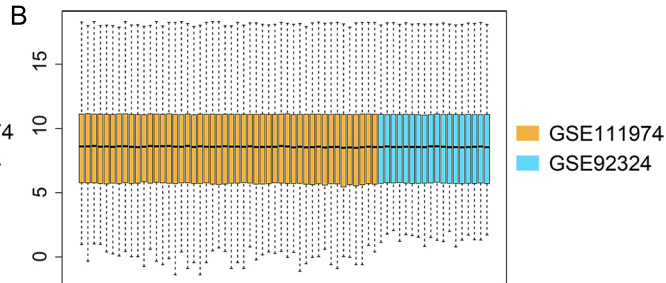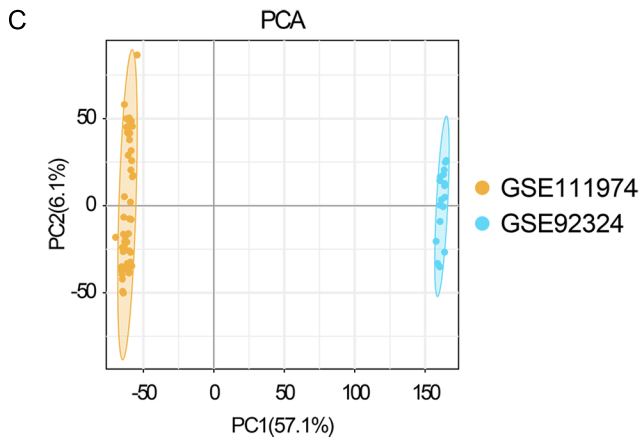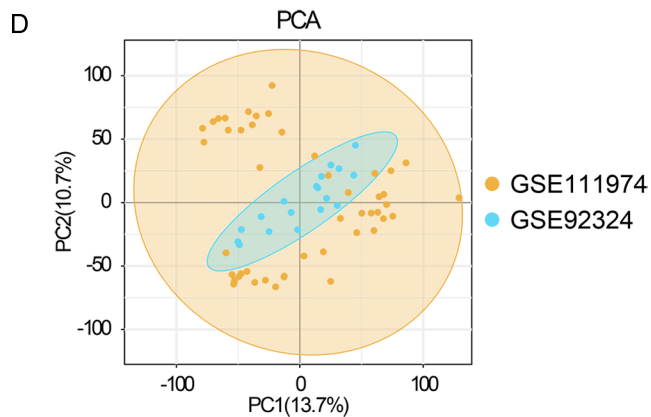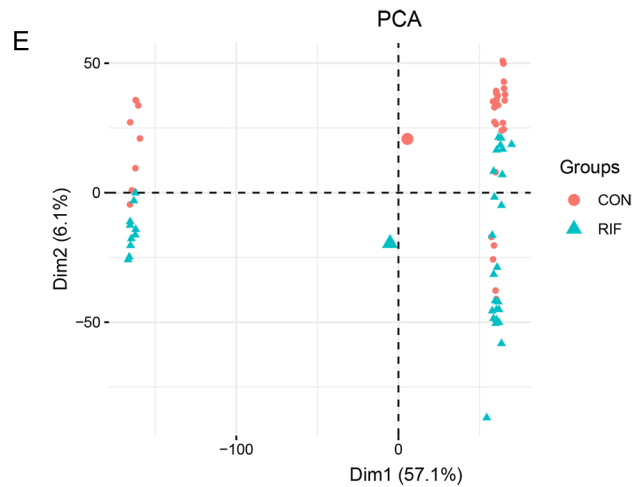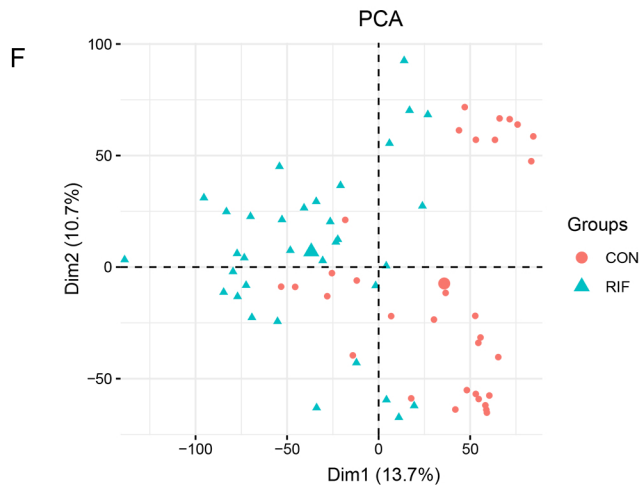

Supplement: Supplementary file 1 [file DataSheet1.ZIP › Figure S2.pdf]

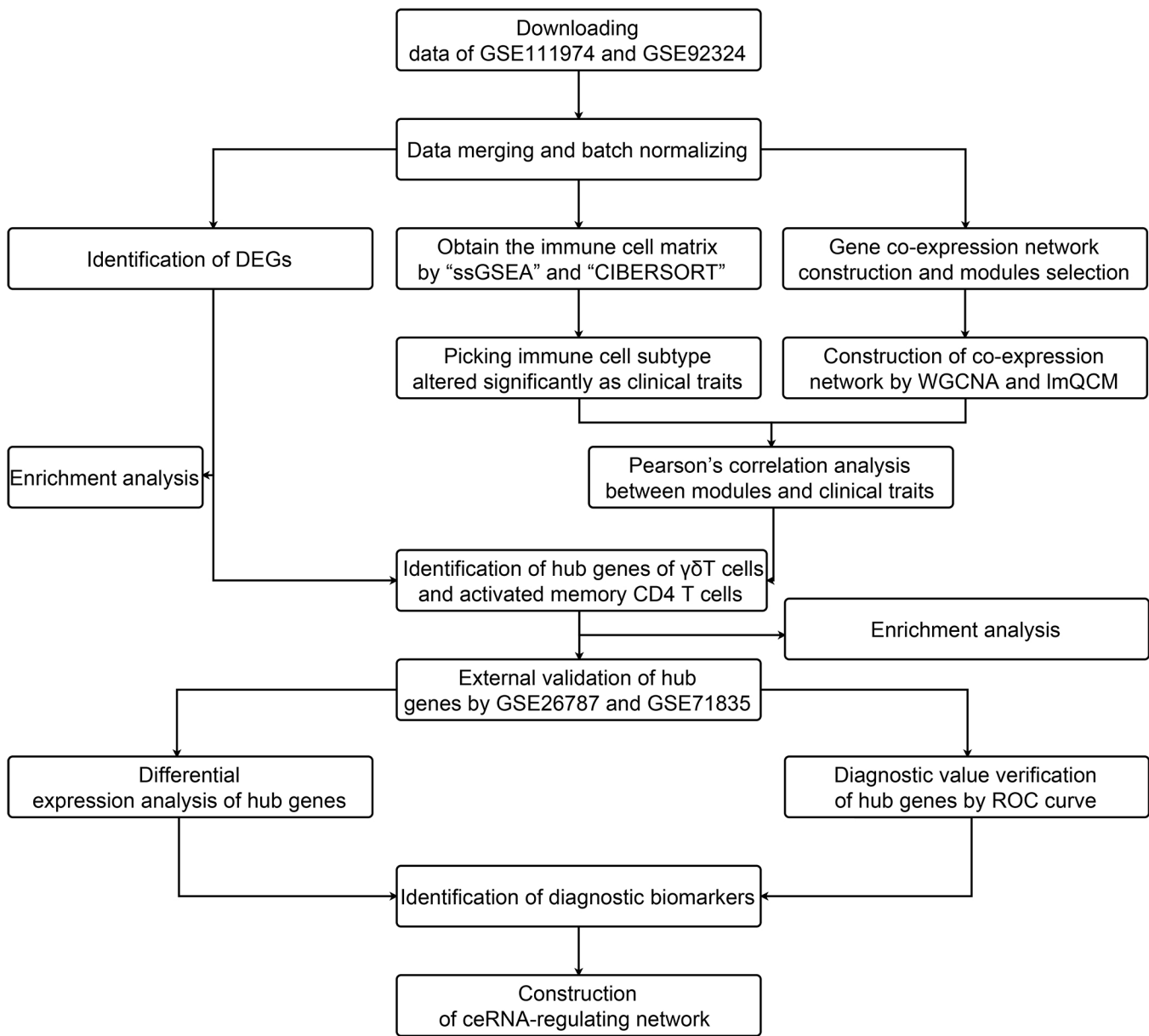

Supplement: Supplementary file 1 [file DataSheet1.ZIP › Figure S3.pdf]

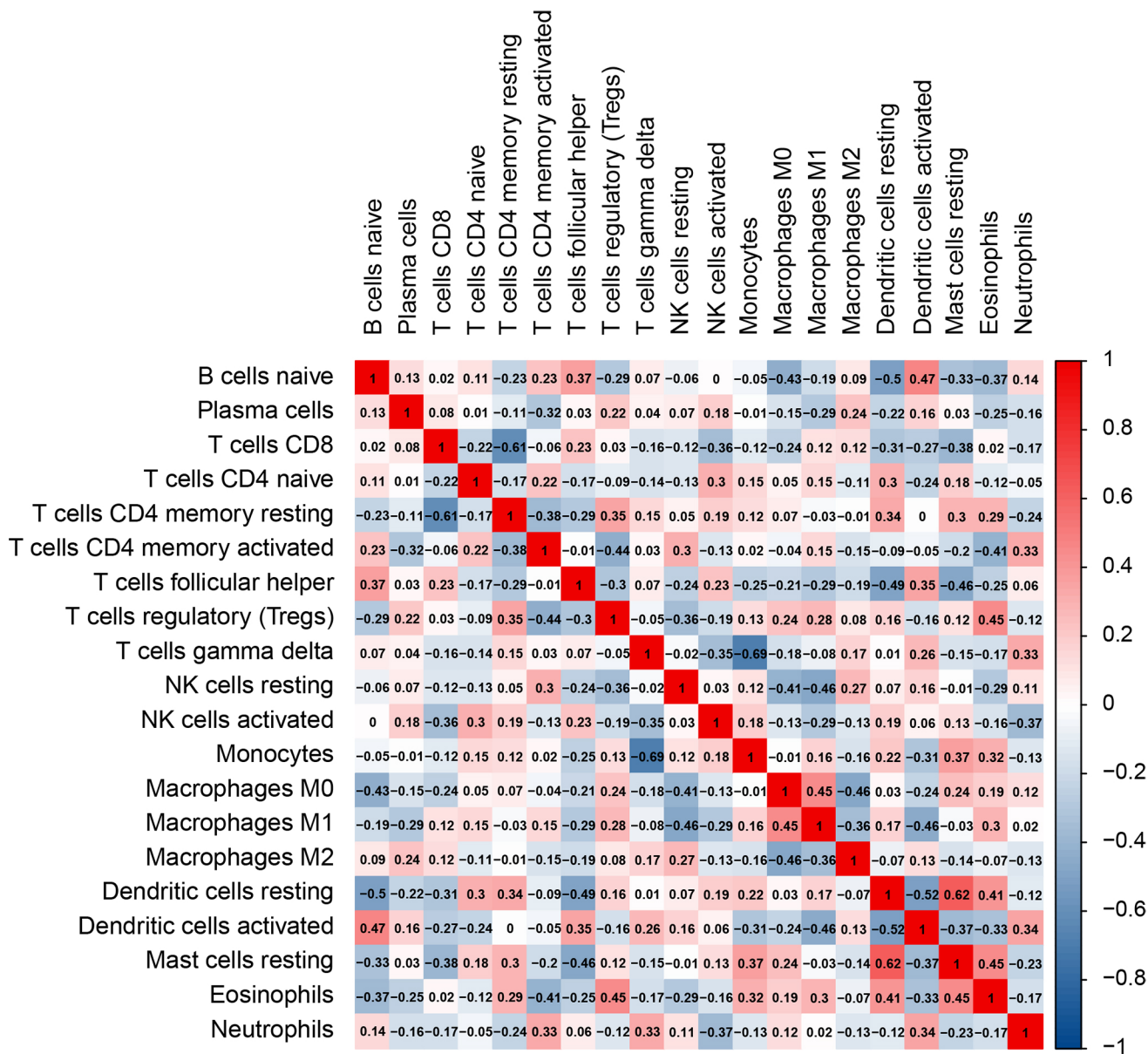

Supplement: Supplementary file 1 [file DataSheet1.ZIP › Figure S4.pdf]

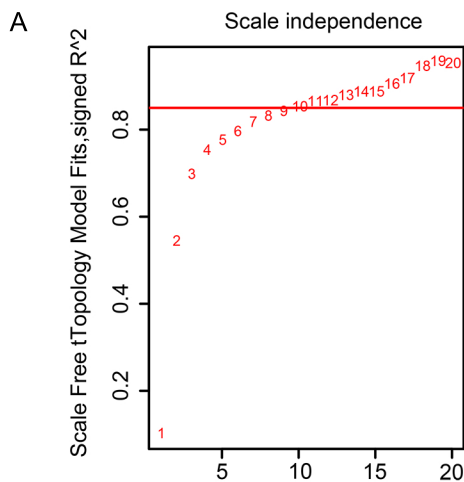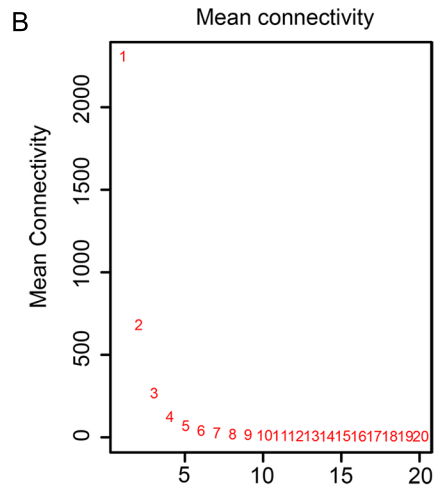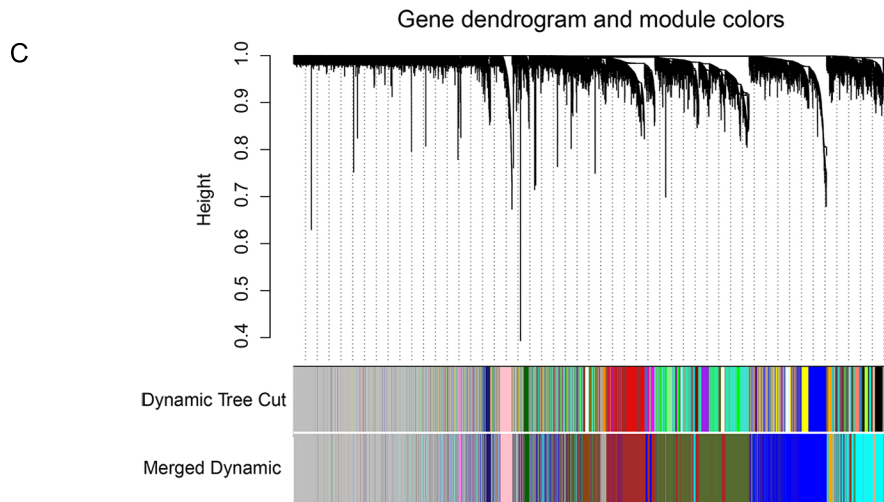

Supplement: Supplementary file 1 [file DataSheet1.ZIP › Figure S5.pdf]

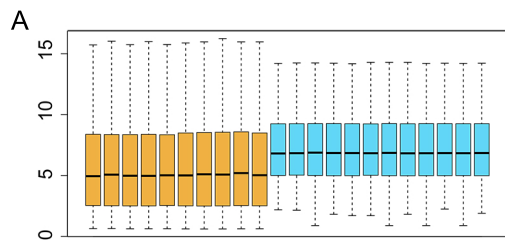

■ GSE26787  
■ GSE71835

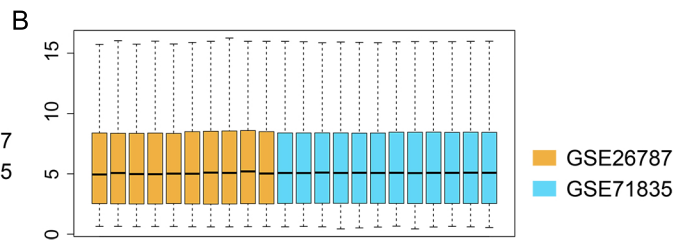

■ GSE26787  
■ GSE71835

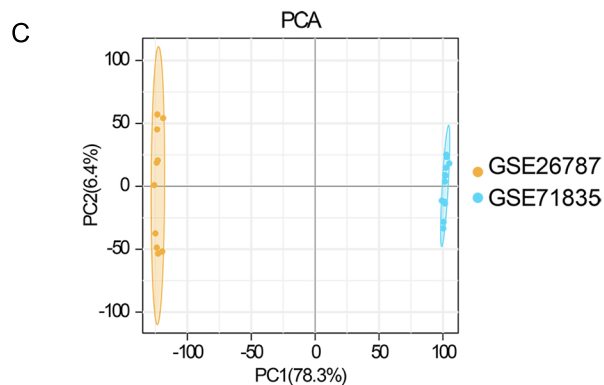

● GSE26787  
● GSE71835

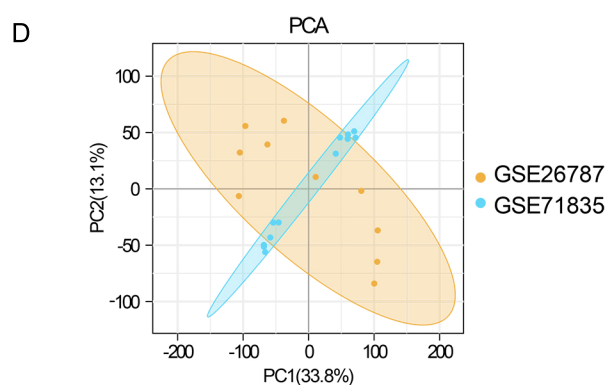

● GSE26787  
● GSE71835

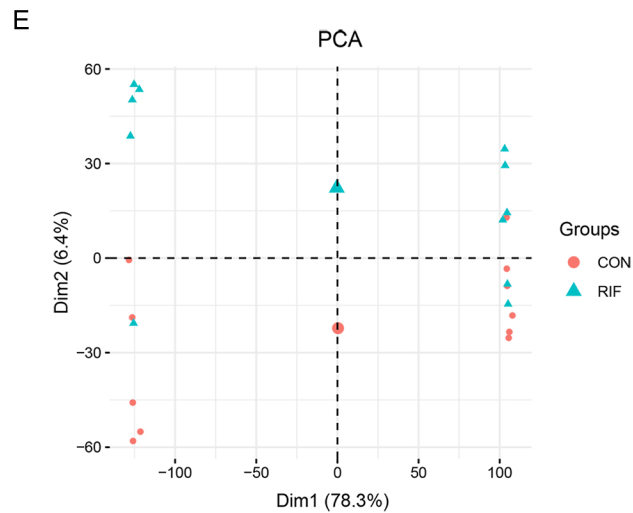

Groups  
● CON  
▲ RIF

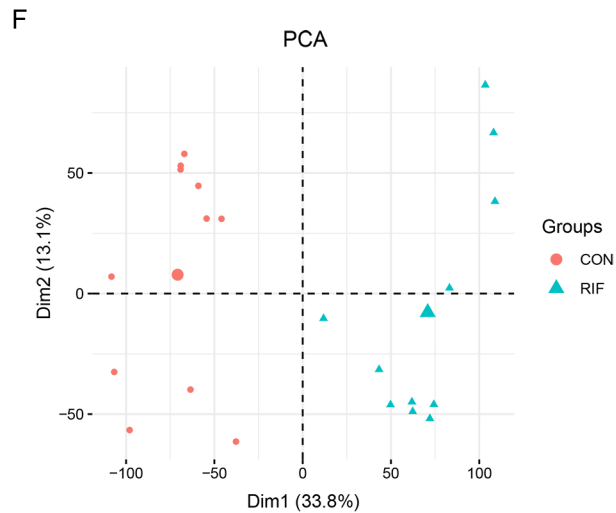

Groups  
● CON  
▲ RIF

Supplement: Supplementary file 1 [file DataSheet1.ZIP › Figure S6.pdf]
